# Effect of Chinese herbal medicine on kinetics of plasma phenylalanine, tyrosine and whole body protein synthesis in sheep

**DOI:** 10.1111/asj.13180

**Published:** 2019-02-17

**Authors:** Xi Liang, Xue Bi, Mohammad Kamruzzaman, Hiroaki Sano

**Affiliations:** ^1^ Department of Animal Science Faculty of Agriculture Iwate University Morioka Japan; ^2^ Present address: Heilongjiang Institute of Veterinary Drug and Feed Control Harbin China

**Keywords:** [^2^H_2_]tyrosine, [^2^H_5_]phenylalanine, Chinese herbal medicine, protein synthesis, sheep

## Abstract

The aim of this study was to evaluate the effect of feeding decoction of a traditional nourishing Chinese herbal medicine formula on rates of plasma phenylalanine and tyrosine turnover and whole body protein synthesis in sheep. Ruminal fermentation characteristics and blood metabolites were also determined. Six sheep were subjected to either mixed hay (MH‐diet, as control) or MH‐diet supplemented with 2% of Chinese herbal medicine (mixture of Astragalus root, Angelica root, and Atractylodes rhizome; CHM‐diet) in a crossover design for each of 3‐week period. The isotope dilution of [^2^H_5_]phenylalanine and [^2^H_2_]tyrosine was performed as a primed‐continuous infusion to measure plasma phenylalanine and tyrosine kinetics. Concentrations of total volatile fatty acid, acetate, and propionate in the rumen tended to be higher (*p* < 0.10), and the pH value was lower (*p* = 0.04) for the CHM‐diet than the MH‐diet. Turnover rates of plasma phenylalanine and tyrosine tended to be higher (*p* < 0.10) for the CHM‐diet than the MH‐diet. Furthermore, whole body protein synthesis was greater (*p* = 0.04) for the CHM‐diet compared with the MH‐diet. The Chinese herbal medicine improved rumen fermentation and enhanced protein metabolism in sheep. Hence, it is suggested that the decoction of Chinese herbal medicine formula could be considered as a potential feed additive for ruminant production.

## 1 INTRODUCTION

The overuse of antibiotics in agricultural industry has received negative social feedback due to the public concern about the hidden danger of drug residues to human health (Khachatourians, 1998). At present, the use of antibiotics as growth promoter in animal diets is restricted in the European Union, Japan, and Korea. It seems likely that the banning policy will spread to the rest of the world within the next few years. Consequently, animal scientists are becoming increasingly concerned about the risk of residual medication and have accelerated to explore potential alternatives to antibiotics for the livestock industry.

Natural herbs have been quickly accepted as a promising alternative to antibiotics for livestock production during the past two decades. The use of numerous herbs as growth promoter or immuno‐potentiator showed great potentials in farm animals (Guo, Kwakkel, Soede, Williams, & Verstegen, 2004; Lien, Horng, & Wu, 2007; Qiao et al., 2012). The mixture of Astragalus root (*Astragalus membranaceus*), Angelica root (*Angelica sinensis*), and Atractylodes rhizome (*Atractylodes lancea*) is a herbal formula for nourishment purpose in traditional Chinese medicine. The herbs contain two main bioactive components, polysaccharides (Astragalus root) and essential oils (Angelica root and Atractylodes rhizome). The health beneficial activity of the components is to restore energy balance by inducing hematopoiesis. In humans, the Chinese herbal formula is used to remove tiredness and comfort stress, which exhibits benefit on maintaining health rather than treating a particular disease or medical condition.

So far, to our knowledge, information is scanty regarding the performance of Chinese herbal medicine on nutrients metabolism in ruminants. We intended to study the effect of the mixture of Astragalus root, Angelica root, and Atractylodes rhizome on intermediary metabolism of different nutrients in sheep through a couple of experiments to introduce a potential feed source for ruminant production. However, in our previous work, feeding the crude herbs did not influence protein metabolism in sheep (Liang et al., 2013). In the treatment of human diseases, liquid extract is the traditional and commonly used form of Chinese herbal medicine. Herbs are processed by a boiling method in order to remove foreign substances and reduce toxic contents as well as increase therapeutic effects (Li & Wei, 2002). It was expected that after processing, the extract of Astragalus root, Angelica root, and Atractylodes rhizome might be effective to enhance protein metabolism in sheep. Therefore, this study was designed to evaluate the effect of Chinese herbal medicine on kinetics of plasma phenylalanine (Phe), tyrosine (Tyr), and whole body protein synthesis (WBPS) in sheep using an isotope dilution method with [^2^H_5_]Phe and [^2^H_2_]Tyr.

## 2 MATERIALS AND METHODS

### 2.1 Animal care

The use and treatment of experimental animals, including cannulation and blood sampling, were approved by the Iwate University. The whole procedure was carried out in strict accordance with the guidelines set by the Animal Care Committee of Iwate University.

### 2.2 Animals and diets

The Chinese herbal medicine used in the experiment was purchased from a traditional Chinese drug market in China. Three herbs were firstly mixed in proportion as 55% of Astragalus root, 27% of Angelica root, and 18% of Atractylodes rhizome according to the formulation of classical Chinese pharmacopoeia. Then the mixture was boiled for three times (20 min for each time) and the boiled water was taken as the extract for use.

Six crossbred (Corriedale × Suffolk) shorn wethers, aged 3 years on average, weighting 46 ± 2 kg, were used in the experiment. Two dietary treatments were tested using a crossover design over two 21‐day periods. The control diet was mixed hay (MH‐diet; dry matter 86.1%, metabolizable energy 1.78 kcal/g, crude protein (CP) 11.7%) of orchardgrass (*Dactylis glomerata*) and reed canarygrass (*Phalaris arundinacea*) offered at maintenance level (NRC, 1985). The experimental diet was the MH‐diet supplemented with 2% of Chinese herbal medicine decoction (CHM‐diet; CP 2.4%). The 2% was based on the original weight of crude herbs and the decoction for feeding was calculated according to the ratio between the amount of herbs and the volume of boiled water (approximately per 308 g to 1 L; 78 ml/head per day). The sheep received mixed hay at 67 g/kg^0.75^ per day in both dietary treatments. During the first 14 days of each experimental period, the sheep were housed in individual pens in an animal barn for adaptation. Then they were moved to metabolic cages and kept in a controlled environment room (23 ± 1°C; lighting from 08:00 to 22:00 hr) for the later 7 days. Three sheep were fed the MH‐diet during the first period and then fed the CHM‐diet during the second period. The other three sheep were subjected to the dietary treatments in the reverse order. The animals were fed twice a day at 08:30 and 20:30 hr with *ad libitum* water access. The extract of Chinese herbal medicine was injected into animal's mouth by a syringe before giving the mixed hay, and they commonly consumed their ration within 1 hr. The sheep were weighted at the start of each dietary treatment and every 7 days intervals.

### 2.3 Rumen fluid collection

On day 20 of each dietary treatment, rumen fluid (30 ml) was taken from each sheep at 0 (before feeding), 3, and 6 hr after feeding via a stomach tube. The pH value was measured immediately after collecting each sample with a pH meter (F‐51; HORIBA, Kyoto, Japan). Then a portion of rumen fluid was centrifuged at 8,000 × *g* for 10 min at 4°C (RS‐18IV; Tomy, Tokyo, Japan) and 1 ml of supernatant was mixed with 1 ml of 0.1 N HCl for ammonia (NH_3_) determination. The prepared samples and the residuals of rumen fluid were stored at −30°C until further analysis.

### 2.4 Isotope dilution method

On day 21 of each dietary treatment, an isotope dilution method using [^2^H_5_]Phe and [^2^H_2_]Tyr was carried out to determine the kinetics of plasma Phe, Tyr, and WBPS in sheep. A catheter for isotope infusion and another for blood sampling were inserted into both jugular veins on the morning of the isotope dilution method. The catheters were filled with sterile solution of 3.8% trisodium citrate. At 12:00 hr, a saline solution (0.9% sodium chloride) containing 2.5 μmol/kg^0.75^ of [^2^H_5_]Phe (L‐phenylalanine, ring‐D5, 98%; Cambridge Isotope Laboratories, Cambridge, MA, USA), 1.6 μmol/kg^0.75^ of [^2^H_4_]Tyr (L‐4‐hydroxypheny1‐2,3,5,6‐D4‐alanine, 98 atom%; Isotec Inc., A Matheson, USA), and 1.6 μmol/kg^0.75^ of [^2^H_2_]Tyr (L‐tyrosine, ring‐3,5‐D2, 98%; Cambridge Isotope Laboratories) was injected as a priming dose through the infusion catheter. Then, [^2^H_5_]Phe and [^2^H_2_]Tyr were continuously infused at constant rates of 2.6 and 1.5 μmol/kg^0.75^ per hour, respectively, for 4 hr using a multichannel peristaltic pump (AC‐2120; Atto, Tokyo, Japan). Blood samples were taken through the sampling catheter immediately before the priming injection (12 ml) and at 30‐min intervals (6 ml) during the last 2 hr of isotope infusion. The collected samples were placed in heparinized tubes and chilled with crushed ice. The catheters were removed after the end of isotope infusion. Plasma was separated from blood samples by means of centrifugation at 10,000 × *g* for 10 min at 4°C and then stored at −30°C.

### 2.5 Analyses

Nitrogen content in diets was analyzed by the Kjeldahl method with a Foss Kjeltec System (Tecator 2520 and Kjeltec 2300; Foss, Hoganas, Sweden). Concentration of NH_3_ in rumen fluid was determined using a colorimetric method (Weatherburn, 1967). Concentrations of volatile fatty acids (VFA) in rumen fluid were measured after steam distillation by a gas chromatography (HP‐5890; Hewlett Packard, Avondale, PA, USA), as reported previously (Sano, Shibasaki, & Sawada, 2009).

Concentrations of plasma free amino acids and urea were determined using the ninhydrin colorimetric method with an automatic amino acid analyzer (JLC‐500/V; JEOL, Tokyo, Japan) as described by Al‐Mamun, Hanai, Tanaka, Tamura, and Sano (2008). Concentration of plasma non‐esterified fatty acid (NEFA) was determined enzymatically with a diagnostic kit (NEFA C; Wako Pure Chemicals, Osaka, Japan). Concentrations of plasma Phe and Tyr, and enrichments of plasma [^2^H_5_]Phe, [^2^H_4_]Tyr, and [^2^H_2_]Tyr were measured according to Rocchiccioli, Leroux, and Cartier (1981) and Calder and Smith (1988) by a gas chromatography mass spectrometry (QP‐2010; Shimadzu, Kyoto, Japan) with selected ion monitoring.

### 2.6 Calculations

The turnover rates of plasma Phe and Tyr (PheTR and TyrTR, respectively) as well as the WBPS were calculated as described by Thompson et al. (1989):

TR=I×(1/E‐1)

where *I* represents the infusion rates of [^2^H_5_]Phe and [^2^H_2_]Tyr, and *E* represents the plasma isotopic enrichments at the steady state. The rate of Phe hydroxylation to Tyr (Phe conversion to Tyr, PheOX) was calculated from the following equation:

$$\text{PheOX}=\text{TyrTR}\;\times \;\left(E_{\mathrm{tyr}}/E_{\mathrm{phe}}\right)\;\times \;\left(\text{PheTR}/\left(I_{\mathrm{phe}}+\text{PheTR}\right)\right)$$

where *E*_tyr_ and *E*_phe_ are the plasma isotopic enrichments of [^2^H_5_]Phe and [^2^H_4_]Tyr, respectively, and *I*_phe_ is the infusion rate of [^2^H_5_]Phe. The WBPS was calculated as follows:

WBPS=(PheTR−PheOX)/Pheconcentrationincarcassprotein

The Phe concentration in carcass protein was estimated to be 35 g/kg (Harris et al., 1992).

### 2.7 Statistics

All data were statistically analyzed with the MIXED procedure of SAS (1996). The analysis of variance was used to test the effects of period and diet, with sheep as the random effect. The significant period effect was not detected in the parameters, therefore only diet effect was considered. Results were defined significant at *p* < 0.05 level, and tendency was at 0.05≤ *p* < 0.10.

## 3 RESULTS

Mean values with standard error of the mean (*SEM*) were given. Body weight gain of sheep and the ruminal fermentation characteristics are shown in Table 1. Body weight gain was similar between dietary treatments. Rumen pH was lower (*p* = 0.04) for the CHM‐diet than the MH‐diet. Concentration of rumen NH_3_ did not differ between diets. Concentrations of rumen total VFA, acetate, propionate, and iso‐valerate tended to be higher (*p* < 0.10) for the CHM‐diet compared with the MH‐diet.

**Table 1:** Effect of Chinese herbal medicine on body weight gain and ruminal fermentation characteristics in sheep^a^

Items	MH‐diet	CHM‐diet	*SEM*	*p*‐value
Body weight gain (kg/d)	0.02	0.03	0.02	0.17
pH	6.78	6.51	0.06	0.04
NH_3_ (mmol/L)	10.8	11.6	0.4	0.19
Total VFA (mmol/L)	83.2	94.4	5.4	0.08
Individual VFA concentrations (mmol/L)				
Acetate	60.3	67.4	2.3	0.07
Propionate	15.7	18.6	0.5	0.08
Iso‐butyrate	0.6	0.6	0.1	0.32
Butyrate	5.3	6.2	0.4	0.21
Iso‐valerate	0.8	0.9	0.06	0.09
Valerate	0.5	0.6	0.03	0.17

*Note*. CHM‐diet, MH‐diet supplemented with 2% of Chinese herbal medicine; MH‐diet, mixed hay of orchardgrass and reed canarygrass; VFA, volatile fatty acids.

a Values represent means and standard error of the mean (*SEM*) of six sheep.

Plasma free amino acids, urea, and NEFA determined in the pre‐infusion period of isotope dilution method are listed in Table 2. Concentrations of plasma valine, leucine, and glycine were lower (*p* < 0.05) for the CHM‐diet than the MH‐diet. Concentrations of plasma threonine, asparagine, and alanine tended to be lower (*p* < 0.10) for the CHM‐diet compared with the MH‐diet. Concentration of plasma urea was similar between diets. Concentration of plasma NEFA was lower (*p* = 0.02) for the CHM‐diet than the MH‐diet.

**Table 2:** Effect of Chinese herbal medicine on concentrations of plasma metabolites at pre‐infusion period in sheep^a^

Items	MH‐diet	CHM‐diet	*SEM*	*p*‐value
Free AA (μmol/L)				
Threonine	274	232	23	0.07
Valine	322	245	21	0.03
Methionine	22	24	2	0.32
Iso‐leucine	101	89	5	0.16
Leucine	151	120	11	0.04
Phenylalanine	49	45	4	0.28
Histidine	57	49	7	0.40
Lysine	132	121	20	0.47
Serine	166	172	19	0.38
Asparagine	62	43	9	0.06
Glutamic acid	91	88	11	0.75
Glutamine	302	321	42	0.61
Glycine	569	503	52	0.04
Alanine	263	210	24	0.09
Tyrosine	66	51	8	0.33
Tryptophan	37	39	7	0.84
Arginine	134	152	10	0.29
Proline	130	107	14	0.19
Urea (mmol/L)	7.5	6.9	0.4	0.56
NEFA (mEq/L)	0.27	0.19	0.02	0.02

*Note*. AA, amino acids; CHM‐diet, MH‐diet supplemented with 2% of Chinese herbal medicine; MH‐diet, mixed hay of orchardgrass and reed canarygrass; NEFA, non‐esterified fatty acid.

a Values represent means and standard error of the mean (*SEM*) of six sheep.

Enrichments of plasma [^2^H_5_]Phe, [^2^H_4_]Tyr, and [^2^H_2_]Tyr were stable during the last 2 hr of isotope infusion (data not shown). Kinetics of protein metabolism measured with the [^2^H_5_]Phe model are presented in Table 3. Concentrations of plasma Phe and Tyr did not differ between dietary treatments. Plasma PheTR and TyrTR tended to be higher (*p* < 0.10), and plasma PheOX was higher (*p* = 0.02) for the CHM‐diet than the MH‐diet. The WBPS was also greater (*p* = 0.04) for the CHM‐diet compared with the MH‐diet.

**Table 3:** Effect of Chinese herbal medicine on kinetics of plasma phenylalanine (Phe), tyrosine (Tyr), and whole body protein synthesis (WBPS) in sheep^a^

Items	MH‐diet	CHM‐diet	*SEM*	*p*‐value
Phe concentration (μmol/L)	33.2	31.9	2.3	0.41
PheTR (μmol/kg^0.75^/hr)	69.0	96.0	12	0.07
PheOX (μmol/kg^0.75^/hr)	8.2	11.9	1.2	0.02
Tyr concentration (μmol/L)	38.4	37.8	3.1	0.53
TyrTR (μmol/kg^0.75^/hr)	59.4	91.4	14	0.06
WBPS (g/kg^0.75^/d)	7.3	10.1	1.4	0.04

*Note*. CHM‐diet, MH‐diet supplemented with 2% of Chinese herbal medicine; MH‐diet, mixed hay of orchardgrass and reed canarygrass; PheOX, rate of phenylalanine hydroxylation to tyrosine; PheTR, turnover rate of plasma phenylalanine; TyrTR, turnover rate of plasma tyrosine.

a Values represent means and standard error of the mean (*SEM*) of six sheep.

## 4 DISCUSSION

### 4.1 Ruminal fermentation characteristics

Essential oils are well known as one of the major bioactive components of Angelica root and Atractylodes rhizome (Wu et al., 2005; Zhou et al., 2012). Essential oils have antimicrobial properties, which could affect rumen VFA concentrations by favorably manipulating microbial fermentation in ruminants (Patra, 2011). In this study, concentrations of rumen total VFA, acetate, and propionate tended to be higher for the CHM‐diet than the MH‐diet. These changes in rumen VFA concentration might be due to the effect of essential oils on rumen fermentation in sheep. The lower rumen pH for the CHM‐diet might be associated with the higher total VFA concentration, which agrees with Allen (1997) who deduced that pH value was inversely related to VFA concentration in the rumen. Although the Chinese herbal medicine showed a positive influence on rumen VFA concentration, rumen NH_3_ remained similar between diets. In ruminants, dietary nitrogenous substances are fermented to NH_3_ in the rumen and the NH_3_ concentration is positively affected by dietary CP intake (Gabler & Heinrichs, 2003; Mahouachi, Haddad, Kayouli, Thewis, & Beckers, 2003). In our study, the two experimental diets were considered to be approximately isonitrogenous due to the low CP content (2.4%) and supplementation level (2%) of Chinese herbal medicine. Hence, similar amounts of nitrogen source should be available for NH_3_ production in the rumen between dietary treatments.

### 4.2 Blood metabolites

Concentrations of numerous plasma amino acids were lower or tended to be lower for the CHM‐diet than the MH‐diet. Generally, a decrease in plasma amino acid concentrations could imply greater utilization for protein synthetic purposes in ruminants (Wessels, Titgemeyer, & St Jean, 1997). Thus, the lower concentrations of certain plasma amino acids suggested a higher incorporation of such amino acids into protein synthesis in sheep. This deduction is in good agreement with the result of WBPS, which was greater for the CHM‐diet compared with the MH‐diet. Plasma NEFA is the best indicator of the actual body lipid loss (Chilliard et al., 2000), which can provide meaningful information regarding negative energy status or stress in ruminants (Fox, Gerrelli, Pitt, & Jacobs, 1991; Hristov et al., 2012). The lower plasma NEFA concentration for the CHM‐diet indicated a reduction in fatty acid mobilization from adipose tissue, which suggested that the bioactive components of Chinese herbal medicine might play a role in regulating energy balance as well as reduce stress in sheep.

### 4.3 Kinetics of protein metabolism

A number of isotope dilution methods using different amino acid isotopes have been applied to estimate the WBPS in experimental animals. Among the isotopes, [1‐^13^C]leucine is the most widely used amino acid isotope (Krishnamurti & Janssens, 1998; Lapierre et al., 2002; Liang et al., 2013). In our current experiment, the isotope dilution method of [^2^H_5_]Phe and [^2^H_2_]Tyr was used to determine protein kinetics in sheep. The WBPS was calculated from the relationship between PheTR and PheOX, where PheOX is the rate of Phe hydroxylation to Tyr. Comparing with the [1‐^13^C]leucine model, the WBPS can be accurately determined within a short time because it does not need to measure the rate of CO_2_ production and the enrichment of exhaled ^13^CO_2_ that derived from leucine oxidation. However, the numerical values of WBPS observed with the [^2^H_5_]Phe model in this study were lower than those observed with the [1‐^13^C]leucine model (calculated from plasma enrichment of α‐[1‐^13^C]ketoisocaproic acid, the true precursor of intracellular leucine metabolism) in sheep in our previous work (Liang et al., 2013). These results accord with the observation of Al‐Mamun, Ito, Sato, Fujita, and Sano (2004) who compared the [^2^H_5_]Phe model with the [1‐^13^C]leucine method to determine protein metabolism in sheep. As expected, the results from this study demonstrated that the Chinese herbal medicine could accelerate turnover and oxidation of plasma amino acids as well as enhance protein metabolism in sheep. The effect of Astragalus root and Angelica root on protein metabolism in nephrotic patients was studied by Li, Yu, and Pan (1995), who used [^15^N]glycine as the tracer and found that Astragalus root and Angelica root could improve the disorder of protein metabolism and increase the net rate of protein synthesis. Zhang, Yang, Wang, and Yang (2013) also reported that the supplementation of Astragalus root powder to basal diet could enhance protein metabolism (increased serum protein) in broiler chickens. Their findings are in good agreement with our present observation in sheep.

## 5 CONCLUSION

In conclusion, the supplementation of Chinese herbal medicine to hay diet was found to improve rumen fermentation and enhance protein metabolism in sheep. It is suggested that the decoction of Chinese herbal medicine formula could be considered as a potential feed source for ruminant production.
